# Draft Genome Sequences of Two Boron-Tolerant, Arsenic-Resistant, Gram-Positive Bacterial Strains, Lysinibacillus sp. OL1 and Enterococcus sp. OL5, Isolated from Boron-Fortified Cauliflower-Growing Field Soils of Northern West Bengal, India

**DOI:** 10.1128/MRA.01438-19

**Published:** 2020-01-09

**Authors:** Subhajit Sen, Tilak Saha, Sabyasachi Bhattacharya, Nidhi, Nibendu Mondal, Wriddhiman Ghosh, Ranadhir Chakraborty

**Affiliations:** a OMICS Laboratory, Department of Biotechnology, University of North Bengal, Siliguri, West Bengal, India; b Laboratory of Immunology, Department of Zoology, University of North Bengal, Siliguri, West Bengal, India; c Department of Microbiology, Bose Institute, Kolkata, India; Indiana University, Bloomington

## Abstract

Two novel boron-tolerant, arsenic-resistant, Gram-positive bacterial strains, Lysinibacillus sp. OL1 and Enterococcus sp. OL5, were isolated from boron fertilizer-amended cauliflower plantation field soils in India. Here, we report the draft genome sequences of OL1 (4.87 Mb) and OL5 (3.93 Mb) to explore the intricacies of boron tolerance in bacteria.

## ANNOUNCEMENT

Boron (B) occurs in soil solution as a nonionized molecule (H_3_BO_3_, a weak monobasic acid) over the pH range optimum for plant growth (1). Boron-tolerant bacteria were isolated from the soil sample of a boron fertilizer-amended cauliflower plantation field (26°31′44.4″N, 88°49′22.8″E) located in Maynaguri, within the Jalpaiguri district of West Bengal, India. A 10-g soil sample was inoculated into 100 ml tryptone soya broth (TSB) supplemented with 50 mM boric acid (H_3_BO_3_) and incubated at 30°C for 72 h. The enriched culture was dilution plated on boric acid-supplemented tryptone soya agar (TSA) for isolation and screening of boron-tolerant strains, as described previously (2). Tolerance of the new isolates toward boron and arsenic, provided as H_3_BO_3_ and sodium arsenite (NaAsO_2_), respectively, was tested in TSA supplemented with different concentrations of boron or arsenic (2); pH tolerance was tested in differentially buffered TSB (3). The intracellular boron concentration was determined by a curcumin-based spectrophotometric method (4) with minor modifications in the process of lysis of the cells grown in boron-supplemented TSB (an ultrasonic homogenizer was used for cell disruption instead of thermal lysis). Two of the new isolates, named OL1 and OL5, retained a significantly lower (≤10%) intracellular boron concentration than that of the medium. OL1 and OL5 tolerated 230 and 210 mM H_3_BO_3_, respectively; the MICs of NaAsO_2_ for these isolates were 6 and 5 mM, respectively. OL1 and OL5 tolerated alkaline conditions up to pH 12 and 10, respectively. The two strains were identified as species of Lysinibacillus and Enterococcus, based on their highest 16S rRNA gene sequence similarities with validly published species (http://www.bacterio.net/), following methods described previously (5).

Genomic DNA was isolated from stationary-phase TSB-grown cells following a standard protocol (6). The DNA was quantified using a SPECTROstar Nano microplate reader (BMG Labtech, Germany). For OL1, a paired-end sequencing library was constructed using a TruSeq Nano DNA library prep kit (Illumina, USA) and sequenced on a NextSeq 500 system (Illumina) using 2 × 150 nucleotides and sequencing by synthesis with dual indexing workflows. This generated 4,872,491 bp of adapter-free (trimming was done with Trimmomatic v0.38, http://www.usadellab.org/cms/?page=trimmomatic) high-quality data, which were subsequently assembled into 179 scaffolds (*N*_50_, 273,708 bp) using SPAdes v3.11.1 with default parameters (7). This sequence data set, as well as the one for OL5 mentioned below, were both quality filtered with a Phred score cutoff of 20 using Fastx_toolkit v0.0.13.2 (http://hannonlab.cshl.edu/fastx_toolkit/download.html). For OL5, a genomic DNA library was constructed using an Ion Xpress Plus fragment library kit (Thermo Fisher Scientific, USA) and sequenced on a 530 chip of the Ion S5 system (Thermo Fisher Scientific). Before sequence retrieval from Ion S5, low-quality and polyclonal reads were filtered out and the adaptor sequences trimmed using the inbuilt PGM software. A total of 3,993,359 bp of adapter-free, quality-filtered reads (mean read length, 277 bp) were obtained and subsequently assembled into 46 contigs (*N*_50_, 211,327 bp) using SPAdes v3.13.0 with default parameters (7); a brief comparison of the two genomes is given in Table 1.

**TABLE 1:** Genomic features and accession numbers of *Lysinibacillus* sp. strain OL1 and *Enterococcus* sp. strain OL5

Strain	Sequencing technology	BioSample accession no.	BioProject accession no.	Assembly accession no.	Genome size (Mb)	Avg genome coverage (×)	G+C content (mol%)	*N*_50_ (bp)	Total no. of genes	No. of coding genes
*Lysinibacillus* sp. OL1	Illumina NextSeq	SAMN10883036	PRJNA521315	GCF_004332175	4.87	287	37.50	273,708	5,008	4,735
*Enterococcus* sp. OL5	Ion S5	SAMN12092207	PRJNA549579	GCF_006491895	3.93	100	41.80	211,327	3,790	3,482

Future comprehensive investigations of the genomics, proteomics, and transcriptomics involving these organisms would illuminate the molecular bases of boron tolerance in the face of alkali challenge.

### Data availability.

These whole-genome shotgun projects have been deposited at DDBJ/ENA/GenBank under the accession numbers SJDS00000000 and VHIL00000000 for *Lysinibacillus* sp. OL1 and *Enterococcus* sp. OL5, respectively. The versions described in this paper are the first versions. The raw sequence reads have been deposited in the Sequence Read Archive (SRA) under the accession numbers PRJNA521315 and PRJNA549579.
